# Autophagy and Diabetic Encephalopathy: Mechanistic Insights and Potential Therapeutic Implications

**DOI:** 10.14336/AD.2021.0823

**Published:** 2022-04-01

**Authors:** Li-zhen Cheng, Wei Li, Yi-xin Chen, Yi-jia Lin, Ya Miao

**Affiliations:** Department of Geriatrics, Shanghai Jiao Tong University Affiliated Sixth People’s Hospital, Shanghai, China

**Keywords:** diabetes mellitus, cognitive impairment, diabetic encephalopathy, autophagy

## Abstract

Diabetic Encephalopathy (DE) is one of the complications of diabetes mellitus (DM) in the central nervous system. Up to now, the mechanisms of DE are not fully discussed by the field. Autophagy is an intracellular degradation pathway crucial to maintain cellular homeostasis by clearing damaged organelles, pathogens, and unwanted protein aggregates. Increasing evidence has demonstrated that autophagy might play an essential role in DE progress. In this review, we summarize the current evidence on autophagy dysfunction under the condition of DE, and provide novel insights of possibly biological mechanisms linking autophagy impairment to DE, as well as discuss autophagy-targeted therapies as potential treatments for DE

Diabetes mellitus (DM) adversely affects multiple organs due to its long-term course of disease, and the brain is one of its major targets. Early in 1922, W.R. Miles and H.F.Root conducted cognitive behavioral tests on 40 diabetic patients, which confirmed that DM caused cognitive impairment [1]. Subsequently, the effects of DM on the central nervous system (CNS) are taken seriously. According to epidemiologic evidence, patients with both type 1 diabetes mellitus (T1DM) and type 2 diabetes mellitus (T2DM) have shown mild-to-moderate reductions in cognitive function as measured by neuropsychological testing compared to non-diabetic controls [2]. In order to promote researches in this field and enhance the understanding of the disease, DeJong first introduced the concept of Diabetic Encephalopathy (DE) [3].

However, as the interaction between DM and CNS is likely to be complex and multifactorial, the mechanisms of DE still remain poorly characterized. Macro-autophagy (herein referred to as autophagy) is a conserved mechanism that cells utilize to degrade intracellular long-lived proteins and organelles through lysosome-mediated degradation. Dysregulation of autophagy has been reported to be linked to a variety of human diseases [4-6]. According to the latest numerous studies, autophagy is also found to be impaired in some regions of brain such as hippocampus and hypothalamus in the condition of DM and deficits in autophagy is likely to involved in the development of DE [7-11].

In this study, we begin with an overview of normal functions of autophagy, and then review the evidence of autophagy dysregulation in DE, followed by the discussion of potential mechanisms associated with autophagy in the pathogenesis of DE. In the end, we enumerate some therapeutic strategies that may be effective in the treatment of DE.

## Autophagy: overview

Autophagy, a catabolic process that removes cell molecules such as protein aggregates and damaged organelles through lysosomal digestion, is essential for intracellular balance [12,13]. There are three major types of autophagy: macro-autophagy, micro-autophagy and chaperone-mediated autophagy (CMA). Macro-autophagy has been regarded as a non-selective cellular process; however, this autophagy controls the quality of cellular contents via selective execution (e.g., long-lived proteins, aggregated proteins, damaged organelles, and intracellular pathogens) [14]. The present review focuses on the mammalian macro-autophagy (hereafter referred to as autophagy).

In general, the autophagy process is summarized as the following steps: 1) induction; 2) autophagosome formation through elongation of an isolated membrane called cisterna; 3) cellular debris sequestration by autophagosome elongation; 4) autolysosome formation as a result of autophagosome-lysosome fusion; 5) digestion of sequestered cargos in the lysosome; 6) maintenance of cellular homeostasis [15]. During the onset of autophagy, several conserved autophagy-related genes (ATG) and their proteins participate sequentially, which is ATG1, ATG17, ATG29, ATG31 and ATG13 in yeast and unc-51 like kinase (ULK)1/ULK2, ATG13, ATG101 and family interacting protein of 200 kDa (FIP200) in mammalians [16].

There are two main pathways involved in the regulation of autophagy. The most well studied pathway is mTOR-dependent signaling pathway, including the AMP-activated protein kinase (AMPK)/mTOR and phosphoinositide 3 kinase (PI3K)/protein kinase B (Akt)/mTOR pathways etc. mTORC1 (mammalian target of rapamycin complex 1) is a complex that mediates the classic functions of mTOR [17,18]. mTORC1 regulates autophagy by controlling the activity of the ULK1 complex. Whenever mTORC1 is activated, autophagy is inhibited due to mTORC1- mediated inhibitory phosphorylation of ULK1. Under diverse cellular stresses, mTORC1 activity is inhibited, thereby promoting ULK1 complex activity and inducing autophagy [19]. mTORC1 can be regulated by diverse signals arising from growth factors (such as insulin), various cytokines, WNT proteins, cellular energy levels [20] etc. Furthermore, autophagy also can be initiated by mTOR-independent signaling pathway, such as Ca^2+^ signal[21], trehalose [22], Ca^2+^ release channel Transient Receptor Potential calcium channel MucoLipin subfamily member 1 (TRPML1)-mediated pathway [23] and so on.

Moreover, microtubule-associated protein 1 light chain 3 (LC3) and sequestosome 1 (p62) are commonly regarded as autophagy-related markers. One approach is to detect LC3 conversion (LC3-I to LC3-II), as the amount of LC3-II is clearly correlated with the number of autophagosomes, LC3-II/LC3-I ratio is generally assumed to be positively correlated with autophagy function. p62 becomes incorporated into the completed autophagosome through its direct binding to LC3 and is subsequently degraded in the autolysosomes; thus, the total cellular expression levels of p62 inversely correlate with autophagic activity [24].

## Evidence of autophagy dysregulation in DE

Multiple lines of evidence indicate autophagy is dysregulated in the context of DM. db/db mice, a monogenic model of T2DM with extreme obesity and hyperglycemia caused by abnormal transcription of leptin receptor protein encoded. In addition, the inbred Goto-Kakizaki (GK) rat is a unique model of spontaneous T2DM caused by naturally occurring genetic variants that have been selectively isolated from an outbred colony of Wistar rats. Above genetic T2DM animal models, showed significantly decreasing abilities in learning and memory as well as impaired autophagy function in the hippocampus, which manifested as a reduction of LC3-II/I ratio and a distinct augment of P62 levels [25-27]. Similar results were obtained in high-fat diet (HFD)/streptozotocin (STZ) injection-induced T2DM C57B/L mice model. Except for performance on behavioral tests was compromised, the level of p62 was increased while the LC3-II/I ratio was decreased in the hippocampus, striatum and hypothalamus of T2DM C57B/L mice [8,9,28]. Moreover, autophagosomal markers (ATG5 and ATG7) which mediate the formation of the autophagosome were decreased in T2DM C57B/L mice, indicating the formation of autophagosome was impaired [28]. Besides, a number of studies showed that T1DM models exhibited autophagy impairment as well. The expression of p62 protein was remarkably elevated while LC3-II/I ratio was abated in the hippocampus of T1DM group, suggesting dysfunction of autophagy flux [7,29,30,31,32]. In vitro studies, compared with the control group, high glucose incubated HT22 cells and primary neurons also showed autophagy was weakened [26,30]. By conducting mRFP-GFP-LC3 puncta analysis, Li et al. [33] demonstrated that autophagic flux was suppressed by high glucose due to impaired autophagosome synthesis.

Interestingly, autophagy dysregulation in the context of DM has been proved playing an important role in DE development. 3-methyladenine (3-MA), a kind of autophagy inhibitor, could further aggravate cognitive impairment in STZ-induced diabetic mice, including exacerbation of anxiety-like behaviors and aggravation in spatial learning and memory, especially the spatial reversal memory [31]. However, other treatments that target autophagy enhancement could alleviate cognitive function in DE models. Study demonstrated that treatment with liraglutide alleviated the learning and memory deficits in diabetic GK rats, particularly in the high-dose liraglutide group, which turns out that liraglutide works by enhancing autophagy [27]. Guan et al. [25] reported administration of granulocyte colony-stimulating factor (G-CSF) significantly improved cognitive function in elderly db/db diabetic mice, and this change was likely related to the improvement of autophagy.

In summary, a number of studies support the argument that autophagy is impaired in DE and based on which, autophagy inhibition can further aggravate cognitive impairment while enhanced autophagy will alleviate symptoms in animal models of DE. Undeniably, autophagy dysfunction plays a crucial role in DE development, we should further explore the potential mechanisms linking autophagy to DE.

## Biological mechanisms linking autophagy and DE

### AD-like pathology and autophagy in DE

Extracellular amyloid plaques formed by β-amyloid peptides (Aβ) and intracellular neurofibrillary tangles (NFTs) consisting of hyperphosphorylated tau protein (p-Tau) are two major pathological hallmarks of Alzheimer's disease (AD). Interestingly, no matter in post-mortem brains of T2DM patients or induction of experimental T2DM models exhibited extracellular Aβ aggregation and intracellular p-Tau deposition [7,34,35,36,37]. Aβ has been demonstrated to be involved in several physiological processes, and an excess accumulation in the brain will lead to neuronal loss and consequently memory deficits[38-40]. In addition, the degree of tau phosphorylation is inversely proportional to tau’s affinity for microtubules and can result in neuronal cytoskeleton destabilization and impaired axonal transport, which in turn can lead to synaptic impairment and progressive neurodegeneration once a pathological threshold is reached [41]. Therefore, the importance of AD-like pathology involved in DE should not be undervalued.

As reported, autophagy is a key regulator of intracellular Aβ clearance. The mechanism of autophagy-mediated clearance involves isolation of cytoplasmic contents by a double-membrane vesicle called an autophagosome or autophagic vacuole (AV). Subsequent lysosomal fusion facilitates degradation of the AV and its contents, which including Aβ and APP-CTFs (amyloid precursor protein-cleaved C-terminal fragment) [42-44]. In addition, autophagy is associated extracellular release of Aβ [45]. As to tau, autophagy is reckoned to be an effective degradation pathway for it [46]. Meanwhile, autophagy also affects the phosphorylation of tau. Inoue et al. [47] found a large amount of highly phosphorylated Tau protein deposits in the brain of mice with ATG7 gene knockout, and this p-Tau deposits would be greatly reduced after autophagy restored. It can be seen that autophagy is pivotal for Aβ and p-Tau metabolism. Once autophagy is impaired, Aβ and p-Tau will deposit in large amounts, which are toxic to brain cells leading to disease development. Unfortunately, Aβ and P-Tau can further provoke defective autophagy, leading to malignant pathological cycles and progression of diseases [48,49].

In recent years, the studies focus on AD-like pathology and autophagy have made a lot of feats in DE. Chen et al. [26] reported enhancing autophagy could reduce tauopathy and thus improve cognitive impairment in db/db mice, so as in high glucose-cultured HT22 cells. In addition, study of Santos et al.[50] observed an increase in tau protein phosphorylation at Ser396 residue in STZ diabetic rats while inducing autophagy partially reversed that effect. Moreover, as reported, the increased Aβ production and cognitive deficits in diabetic mice were reversed by rapamycin (a autophagy activator) treatment [32]. Many other studies have reached similar conclusions [10,51]. Above all, impaired autophagy is a well-established participating mechanism in the AD-like pathology of DE and should not be underestimated.

### Neuroinflammation and autophagy in DE

In general, inflammatory response may serve as a protective mechanism in the brain, however, excessive inflammation can lead to neuronal damage. Many studies reported higher levels of pro-inflammatory factors, such as TNF-α, IL-1β, in different brain tissues of STZ diabetic rats and mice [52-54]. Continuous hyperglycemia under diabetes can trigger activation of the nuclear factor kappa-B (NF-κB) pathway and release of pro-inflammatory factors, resulting in an imbalance between the pro-inflammatory and anti-inflammatory networks, leading to unrestricted formation of inflammatory mediators, thereby causing neuronal damage [55]. In conclusion, diabetes can significantly induce neuroinflammation, which is an important mechanism of DE.

Recently, the role of autophagy involved in inflammation is gradually recognized, which is also one of the important interactions between autophagy and DE. NF-κB is a transcriptional factor that participates in the modulation of inflammation. Some studies have demonstrated the interplay between autophagy and NF-κB signaling pathway. Overexpressed ATG5, but not the autophagy-incompetent ATG5 mutant K130R in HK-2 cells, could suppressed inflammatory response via inhibition of NF-κB signaling [56]. Another study figured out pharmacological administration of mTOR inhibitors and autophagy stimulators markedly ameliorated inflammatory in vivo [57]. In addition, reduced autophagy may also enhances microglia activation, including secretion of pro-inflammatory cytokines such as Il-1β in vitro [58]. Except for the above cases, autophagy has also been reported to regulate inflammation through other pathways [59].

In a study of Cui et al. [28], they found melatonin (MLT) treatment significantly improved neuro-inflammation and ameliorated cognitive impairment in T2DM mice. However, 3-MA treatment increased the neuroinflammation, indicating that the treatment effect of MLT was mediated by autophagy and targeting autophagy enhancement had a therapeutic effect on DE by alleviating neuroinflammation. Summarizing, these studies suggest that autophagy might play a protective role during the course of DE via interaction with neuroinflammation.

### Synaptic plasticity and autophagy in DE

Synaptic plasticity is deemed as the neurobiological basis of learning and memory and has long been one of the hottest pots in molecular and cellular neurobiology. Many studies support that diabetes leads to a significant decrease in the number of hippocampal synapses, synaptic degeneration, blurred synaptic gaps, discontinuous synaptic connections, affecting synaptic function, and leading to spatial learning and memory disorders [60,61]. In addition to changes in synaptic structural plasticity, functional plasticity of hippocampal synapses is also impaired in diabetes. Long-term potentiation (LTP) and long-term depression (LTD), believed to be related to the cellular mechanisms of learning and memory, have been the object of intense investigation of synaptic functional plasticity. In the hippocampus of STZ diabetic rats, the magnitude of higher frequencies-induced LTP is strongly reduced[62,63] whereas that of low frequencies-induced LTD is either increased[64] or not changed [63]. In conclusion, DE is closely related to changes in brain synaptic plasticity.

Many studies indicated autophagy is crucial to synaptic plasticity in neurons, once dysregulated, might contribute to brain disorders. Recent studies have shown that autophagy can selectively target synaptic components, regulate the stability of specific proteins in synapses. One step further, this process is also regulated by neuronal activity, thus contributing to the maintenance of specific functions of neurons [65-67]. In addition, autophagy is also involved in synaptic plasticity in microglia [68].

Above findings suggest that there is a non-trivial connection between synaptic plasticity and autophagy. In a study of DE, LTP and depotentiation (DPT) were exacerbated by autophagic inhibition in diabetic mice, which indicating impairment of synaptic plasticity. However, no significant change of pair-pulse facilitation (PPF) was recorded in diabetic mice with autophagic suppression compared with the diabetic mice, which implied that presynaptic function was not affected by autophagic inhibition in diabetes [31]. Further investigation is required to fully understand the mechanisms involved in the association of synaptic plasticity and autophagy during DE process.

## Oxidative stress and autophagy in DE

In DM, hyperglycemia reduces antioxidant levels and concomitantly increases the production of free radicals, which contributes to tissue damage and leads to alterations in the redox potential of the cell with subsequent activation of redox-sensitive genes [69]. In addition, the brain is particularly sensitive to oxidative damage as a result of its high oxygen consumption rate, abundant lipid content, and relative paucity of antioxidant enzymes as compared to other tissues [70]. A number of studies provided the evidence that the activities of superoxide dismutase (SOD), catalase and antioxidant enzymes were significantly decreased in the brain of DM models [71-74].

As reported, when reactive oxygen species (ROS) causes intracellular energy imbalance, ROS will activate AMPK [75], and then through AMPK/tuberous sclerosis complex (TSC) 1/TSC2/Rheb (a Ras-related GTP binding protein ) pathway to inhibit mTOR and finally promote autophagy activation [76]. Activated autophagy is a response to oxidative stress and protects the cells from apoptosis[77], whereas impairment of autophagy will cause accumulation of oxidative stress [78]. Kelch-like ECH associated protein 1 (Keap1)/nuclear factor erythroid-2-related factor 2 (Nrf2)/antioxidant response element (ARE), one of the most important antioxidant pathways, is substantial for resisting ROS-induced damage. Interestingly, P62, as an autophagy substrate, can regulate the Keap1/Nrf2/ARE pathway. Phosphorylated P62 can increase the activity of binding to Keap1, while Keap1 loses its chance to bind to NRF2, then the NRF2 can enter into the nucleus and participate in the regulation of ARE antioxidant elements [79]. As previous study reported, upregulation of autophagy by rapamycin decreased oxidative stress-induced generation of ROS, whereas inhibition of autophagy by 3-MA or by knockdown of ATG7 or BECN1 increased ROS generation, exacerbated oxidative stress-induced reduction of mitochondrial activity, reduced cell viability [80].

Fakih et al. [81] discovered treatment with metformin or pioglitazone in non-obese rat model of prediabetic could reversed oxidative stress markers ameliorated cognitive function, which was owing to the autophagy activation. In addition, following the administration of 5-PAHSA (a novel palmitic acid hydroxy stearic acid) in PC12 cells under diabetic conditions, increased levels of autophagy were observed and the concentration of ROS declined, which implied a neuroprotective role [82]. Zucker Diabetic Fatty (ZDF) rat, a model of T2DM, is characterized by a mutated leptin receptor gene. Talaei et al.[83] observed ZDF brains showed a higher level of reactive oxygen species. Treatment of ZDF brain slices with NaHS, however, enhanced autophagy while counteracting oxidative stress. From the above studies, we can infer that abnormal autophagy in DM is closely related to oxidative stress at the organ level, which makes us full of imagination about the aggravation of oxidative stress caused by down-regulation of autophagy in DE.

## Potential Therapeutic Implications of autophagy in DE

In recent years, powered by drug structure and more thorough research on the molecular mechanism and related studies on DE, the studies on DE related therapies have also made some progress. The potential therapeutic implications on DE by targeting autophagy are summarized as follows and presented in detail in Table 1.

**Table 1:** Potential therapeutic implications on DE by targeting autophagy.

Classification	Name	Dosages	Effects on DE	Molecular mechanisms	Models	Refs
Antidiabetic drugs	Liraglutide	37.5, 75, 150, 200 µg/kg	Decrease diabetes-induced cell loss and pyknosis, improve cognitive function	Induce autophagy by activating AMPK/mTOR and PI3K/Akt/mTOR signaling	GK rat	[27]
		200 mg/kg/day for 8 weeks, i.p. injection; 100 nM for 24 h	Improve cognitive function	Induce autophagy by activating AMPK/mTOR signaling	STZ-induced mouse model of T1DM; high glucose-treated hippocampal primary neurons	[86]
	Ex-4	5 μg/kg/day for 28 days, infusion rate 2.5 μL/h	Inhibit cell apoptosis	Induce autophagy by increasing PI3K class III	GK rat	[87]
	Metformin	200 mg/kg/day for 8 weeks, i.p. injection; 3.2 mM for 24 h	Decrease p-Tau burden and improve cognitive function	Induce autophagy by AMPK dependent pathway	db/db mice; high glucose-cultured HT22 cells	[26]
	Insulin	2U/day (blood glucose levels were <= 200 mg/dL), an extra 2U per each 100 mg/dL blood glucose were given if blood glucose levels were > 200 mg/dL, for one month, s.c. injection	Decrease p-Tau burden	Induce autophagy by inhibiting mTOR activity	STZ-induced rat model of T1DM	[50]
Herbal medicine	HGSD	40 mg/kg; 2.5, 5, 10 μm incubate for 48 h	Inhibit neuronal apoptosis	Induce autophagy by activating AMPK/mTOR signaling	HFD/STZ-induced mouse model of T2DM; high glucose-treated SH-SY5Y cells	[92]
	ZBPYR	0.4637 g of the raw medicinal herbs in 0.14 ml water per 10 g of body weight for 15 days	Decrease Aβ burden and improve cognitive function; Inhibit cell apoptosis	Induce autophagy by inhibiting mTOR/p70S6K signaling	ZDF rats	[51]
	NC	100 mg/kg/day, for 35 consecutive days, i.p. injection	Attenuate neuronal loss as well as cellular ultrastructure impairment and improve cognitive function	Induce autophagy by inhibiting mTOR/p70S6k signaling	HFD/STZ-induced rat model of T2DM	[29]
Other chemicals	Rapamycin	8.5 mg/kg/day for 5 consecutive days, i.p. injection; 200 nM for 30 min	Decrease Aβ burden and improve cognitive function	Induce autophagy by activating AMPK/mTOR signaling	STZ-induced mice model of T1DM; high glucose-cultured human neuroblastoma cell line SK-N-MC	[32]
	Melatonin	10 mg/kg/day for 1 month; 100 nM for 24 h	Inhibit neuroinflammation and improve cognitive function; inhibit cell apoptosis	Induce autophagy by inhibiting TLR4/Akt/mTOR pathway	HFD/STZ-induced mouse model of T2DM; palmitic acid-stimulated BV-2 cells	[28]
	G-CSF	75 μg/kg for 1 month, i.p. injection	Improved brain activity as well as the connectivity of the hippocampus and improve cognitive function	Induce autophagy by enahcing Beclin1 and inhibiting mTOR activity	db/db mice	[25]
	5-PAHSA	50, 150 mg/kg/day for 30 days, oral gavage	Reduce oxidative stress	Induce autophagy by activating ULK1/mTOR signaling	db/db mice; high glucose-cultured PC12 cells	[82]
	L-NNA	0.2 mM	Decrease numbers of propidium iodide-positive cells	Induce autophagy by abolishing ATG4B S-nitrosation	High-glucose-treated SH-SY5Y cells	[33]
	NaHS	50 μM every 10 h for 2 days	Decrease p-Tau burden and reduce oxidative stress	Induce autophagy by inhibiting mTOR activity	Cultured brain slices from ZDF rats	[83]
	Meloxicam	1, 3 mg/kg	Decrease Aβ burden and improve cognitive function	Induce autophagy by inhibiting COX2/PGD2 signaling	HFD/STZ-induced rat model of T2DM	[10]
Genetic targets	TIGAR	overexpression of TIGAR by lentivirus	Improve cognitive function; inhibit cell apoptosis	Induce autophagy by inhibiting NOS1	STZ-induced mouse model of T1DM; high-glucose-treated hippocampal primary neurons	[30]
	MEG3	overexpression of MEG3 by lentivirus	Reduce oxidative stress and improve cognitive function; inhibit cell apoptosis	Promote FUNDC1-Related Mitophagy via Rac1-ROS Axis	STZ-induced mouse model of T1DM; high-glucose-treated PC12 cells	[101]

Ex-4, exendin- 4; HGSD, Huang-Gui Solid Dispersion; ZBPYR, ZiBuPiYin recipe; NC, Nicotinate-curcumin; G-CSF, granulocyte colony-stimulating factor; L-NNA, Nv-nitro-L-arginine; MEG3, maternally expressed gene 3; i.p. injection, intraperitoneal injection; s.c. injection, subcutaneous injection

### Antidiabetic drugs

Glucagon-like peptide-1 (GLP-1) receptor agonists are a novel type of antidiabetic drug used in clinical practice, which have been proved to possess numerous potential benefits in improving capacity in many diabetic complications by targeting autophagy [84,85]. It has also been reported in studies of DE. Liraglutide, a type of GLP-1 receptor agonist, was figured out could decrease diabetes-induced cell loss and pyknosis and improve cognitive function that acts by increasing mTOR expression via the AMPK and PI3K/Akt pathways [27]. In addition, Kong et al. [86] reported liraglutide promoted autophagy as indicated by enhanced expression of the autophagy markers LC3-II and Beclin 1, decreased expression of p62, and increased formation of autophagic vacuoles and LC3-II aggregates. Meanwhile, liraglutide exhibited neuroprotective effects against diabetes-induced hippocampal neuronal injuries and cognitive impairment via the AMPK/mTOR pathway. Besides, exendin-4 (Ex-4), a kind of GLP-1 mimetics, was also indicated constituting a promising therapy against the chronic complications of T2DM affecting the brain by inducing autophagy through increasing PI3K class III [87].

Metformin is one of the first-line treatments for glycemic control in T2DM patients. Interestingly, beyond its glucose-lowering effect, metformin possesses a variety of other beneficial effects according to emerging clinical and experimental studies, which is mostly due to its function in mediating autophagy activation[88,89]. In a study of Chen et al. [26],12-week-old male db/db mice received consecutive intraperitoneal injection of 200 mg/kg/d metformin or (and) 10 mg/kg/d chloroquine for eight weeks. Metformin attenuated cognitive impairment in db/db mice, reduced p-Tau proteins, restored the impaired autophagy in diabetic mice, all of which were reversed by chloroquine (an autophagic flux inhibitor) treatment via inhibiting of autophagy activity. In high glucose-cultured HT22 cells, metformin enhanced autophagy in a dose-dependent manner. Mechanically, it is proved that metformin enhanced autophagy activity in an AMPK dependent manner.

T1DM patients critically depend on lifelong insulin treatment to survive. On top of that, insulin therapy is expected to control some of the long-term complications associated with extremely high levels of blood glucose under T1DM condition [90]. Interestingly, Santos et al.[50] demonstrated daily subcutaneous injections of insulin could dwindle p-Tau burden of STZ-induced rat model of T1DM, which ascribed to enhanced autophagy function by inhibiting mTOR activity. Briefly, insulin treatment is capable to normalize the alterations induced by T1DM supporting the importance of autophagy signaling.

Altogether, in addition to controlling blood glucose, antidiabetic drugs administration may also be a promising therapy against the chronic complications of diabetes affecting the brain.

### Herbal medicine

Herbal medicine has been used for the treatment of diabetes and dementia for thousands of years in China. Modern pharmacological research uncovers that some active components from herbal medicine exert curative effect on DE by targeting autophagy signaling. Huang-Gui Solid Dispersion (HGSD), developed to improve oral bioavailability of Berberine (BBR), was indicated exhibited a good hypoglycemic activity by promoting AMPK activation[91]. Additionally, Xue et al. [92] figured out HGSD significantly inhibited cell apoptosis, enhanced cell autophagy and activated the AMPK/mTOR pathway in the hippocampus of diabetic mice. Moreover, HGSD vastly attenuated apoptotic death, enhanced autophagy and activated the AMPK/mTOR pathway in high glucose-treated SH-SY5Y cells. Accordingly, HGSD could protect against neurotoxicity induced by high glucose through activating autophagy and eventually inhibiting neuronal apoptosis, which was activated by the AMPK/mTOR signaling pathway.

ZiBuPiYin recipe (ZBPYR), a traditional formula of Chinese medicine documented in the book of Bujuji written by Wu Cheng in the Qing dynasty, is derived from Zicheng Decoction and used for the treatment of cognitive impairment. Early reports have indicated that ZBPYR improved the learning and memory process in HFD/STZ-induced rat model of T2DM, and regulated the deposition of Aβ in the brain [93]. Recently, Bi et al. [51] confirmed ZBPYR treatment considerably reduced the deposition of Aβ, and ameliorated learning and memory impairments of ZDF rats with chronic psychological stress, which might contribute to inducing autophagy by inhibiting mTOR/p70S6K signaling.

Curcumin, a major bioactive component of turmeric, has various pharmacological activities including regulation of autophagy [94]. Nicotinate-Curcumin (NC), a novel curcumin derivative derived from nicotinate and curcumin, which has superior water solubility and bio-availability[95]. Study of Gu et al. [29] showed that NC treatment improved cognitive deficit, attenuated neuronal loss and cellular ultrastructure impairment in the CA1 region of T2DM rats. Notably, NC treatment reversed autophagic flux impairment as evidenced by the deceases in LC3-II and p62 protein levels, and autophagosome accumulation in the hippocampal CA1 region of T2DM rats. However, these protective effects of NC were diminished by cotreatment with 3-MA and chloroquine, respectively. These results indicate that NC ameliorates diabetes-induced cognitive function impairment via restoring autophagic flux.

In a nutshell, herbal medicine may be a promising agent for DE prevention and treatment through autophagy activation. Therefore, identifying natural products of autophagy regulators is of remark importance.

### Other chemicals

Rapamycin, a well-known autophagy activator, through selectively inhibiting of mTORC1 and thus modulator of the autophagy activity. It has been proved playing a role in improving learning and memory and reducing Aβ and P-tau pathology in the brains of AD mouse model via enhancing autophagy activity [96,97]. Chae et al. [32] reported rapamycin lessened Aβ burden and prevented cognitive impairment in STZ-induced mice model of T1DM, which dues to autophagy enhancement by activating AMPK/mTOR signaling.

Melatonin (MLT) is the main secretory product of the pineal gland. It acts as a regulator of the circadian rhythm. A recent study indicated MLT administration significantly improved neuroinflammation and regulated microglial apoptosis. Furthermore, 3- MA increased the microglial inflammation and apoptosis, indicating that the treatment effect of MLT was mediated by autophagy [28].

G-CSF is a glycoprotein that promotes the production of granulocytes and stem cells in bone marrow as well as their release into the blood stream, which aids in the proliferation and differentiation of neurotrophic factors [98]. Guan et al. [25] figured out administration of G-CSF significantly improved cognitive function in elderly db/db diabetic mice, and this change was likely related to the regulation of autophagy.

In addition, 5-PAHSA [82], Nv-nitro-L-arginine (L-NNA)[33], NaHS[83] and meloxicam[10] were also revealed as potential strategy against DE via up-regulation of autophagy.

### Genetic targets

TP53-inducible glycolysis and apoptosis regulator (TIGAR) is an endogenous inhibitor of glycolysis and increases the flux of pentose phosphate pathway (PPP) by regulating glucose 6-phosphate dehydrogenase (G6PD). TIGAR is highly expressed in neurons, and its role in DE was revealed recently. As reported, TIGAR was decreased in the hippocampus of STZ-induced diabetic mice as well as high-glucose-treated hippocampal primary neurons. Importantly, overexpression of TIGAR ameliorated STZ-induced cognitive impairment in mice and reduced cell apoptosis. Furthermore, enhancing the expression of TIGAR rescued high glucose-induced autophagy impairment. Nitric oxide synthase 1 (NOS1), a negative regulator of autophagy, was also inhibited by overexpression of TIGAR. However, inhibition of autophagy abolished the protective effect of TIGAR [30]. Therefore, TIGAR may have a therapeutical effect via up-regulation of autophagy in DE.

In recent years, long noncoding RNAs (lncRNAs) have been identified to be associated with diabetes and its complications [99]. Maternally expressed gene 3 (MEG3), a lncRNA gene localized at chromosome 14q32, is widely expressed in several normal tissues [100]. Wang et al. [101] observed that MEG3 was significantly down-regulated in STZ-induced diabetic rats. MEG3 overexpression noticeably improved diabetes-induced cognitive dysfunctions, accompanied by promoting Mitophagy. Conversely, knockdown of MEG3 showed opposite effects.

However, autophagy associated genetic targets in DE, especially microRNAs (miRNAs) and transcription factors, are so far not fully understood and needs further investigation.

## Conclusion and prospect

A growing number of studies point to the dysregulation of autophagy in DE, which is associated with DE progressing. In addition, the autophagy plays a key role in AD-like pathology, neuroinflammation, synaptic plasticity and oxidative stress, all of which may mediate its effect on DE. According to previous studies, DE related therapies by targeting autophagy have also made some progress. However, such autophagy-targeted therapy applying for DE remained poorly investigated. Therefore, a deeper and more comprehensive understanding of the autophagy-targeted therapeutic approaches in DE is needed in the near future.
